# High frequency of lactose intolerance in a prehistoric hunter-gatherer population in northern Europe

**DOI:** 10.1186/1471-2148-10-89

**Published:** 2010-03-30

**Authors:** Helena Malmström, Anna Linderholm, Kerstin Lidén, Jan Storå, Petra Molnar, Gunilla Holmlund, Mattias Jakobsson, Anders Götherström

**Affiliations:** 1 Department of Evolutionary Biology, Uppsala University, 752 36 Uppsala, Sweden; 2 Archaeological Research Laboratory, Stockholm University, 106 91 Stockholm, Sweden; 3 Osteoarchaeological Research Laboratory, Stockholm University, 106 91 Stockholm, Sweden; 4 National Board of Forensic Medicine, Department of Forensic Genetics and Forensic Toxicology, 587 85 Linköping, Sweden; 5 Current address: Department of Archaeology, University College Cork, Cork, Ireland

## Abstract

**Background:**

Genes and culture are believed to interact, but it has been difficult to find direct evidence for the process. One candidate example that has been put forward is lactase persistence in adulthood, i.e. the ability to continue digesting the milk sugar lactose after childhood, facilitating the consumption of raw milk. This genetic trait is believed to have evolved within a short time period and to be related with the emergence of sedentary agriculture.

**Results:**

Here we investigate the frequency of an allele (-13910*T) associated with lactase persistence in a Neolithic Scandinavian population. From the 14 individuals originally examined, 10 yielded reliable results. We find that the T allele frequency was very low (5%) in this Middle Neolithic hunter-gatherer population, and that the frequency is dramatically different from the extant Swedish population (74%).

**Conclusions:**

We conclude that this difference in frequency could not have arisen by genetic drift and is either due to selection or, more likely, replacement of hunter-gatherer populations by sedentary agriculturalists.

## Background

The ability to drink milk as an adult occurs at a high frequency in present-day populations that practice dairying and cattle rearing [1-4]. It has been suggested that this correlation represents a case of gene-culture co-evolution, i.e. an adaptive genetic trait exposed to positive selection induced by cultural practices. The -13910*T allele associated with this trait in Europeans appears to have been the target of strong selection over a relatively short period of time [5,6]. Such selection pressure could be one of several explanations for the high frequency of the derived allele (associated with allowing milk consumption in adulthood) in northern Europeans, the region where the allele is most common (74% in Sweden [7]). A single nucleotide polymorphism (SNP-13910 T/C), strongly associated with the ability to digest lactose in adulthood [8], has been used as a marker for the genetic trait. Notably, the allele frequency at the SNP and the extent of haplotype homozygosity around the particular SNP-allele indicate a history of a strong positive selection [5,6,9,10], and it has been suggested that this selective pressure was attributable to the introduction of agriculture and animal domestication [10,11].

The Pitted Ware Culture (PWC) was a major Neolithic hunter-gatherer population in Northern Europe and was partly contemporaneous with the farming TRB population (TRB after the German word Trichterbecherkultur, i.e Funnel Beaker Culture). The PWC are thought to have been present in Scandinavia between 5,400-4,300 years before present (BP) [12], which is later than the suggested initiation of selection for the T allele [6]. In this study, we find that the frequency of the derived allele is low in the PWC (5%) compared to the frequency in the extant Swedish population, and that the change in frequency is incompatible with genetic drift as the sole explanation under a model of population continuity. Thus, a genetic component interacting with culture, such as the ability to digest milk as an adult, could have been the result of the replacement of the hunter-gatherer population by an agricultural population. Alternatively, positive selection could have dramatically increased the frequency of the derived allele in the PWC, allowing for population continuity from the PWC to the extant Swedish population.

## Methods

**Figure 1:**
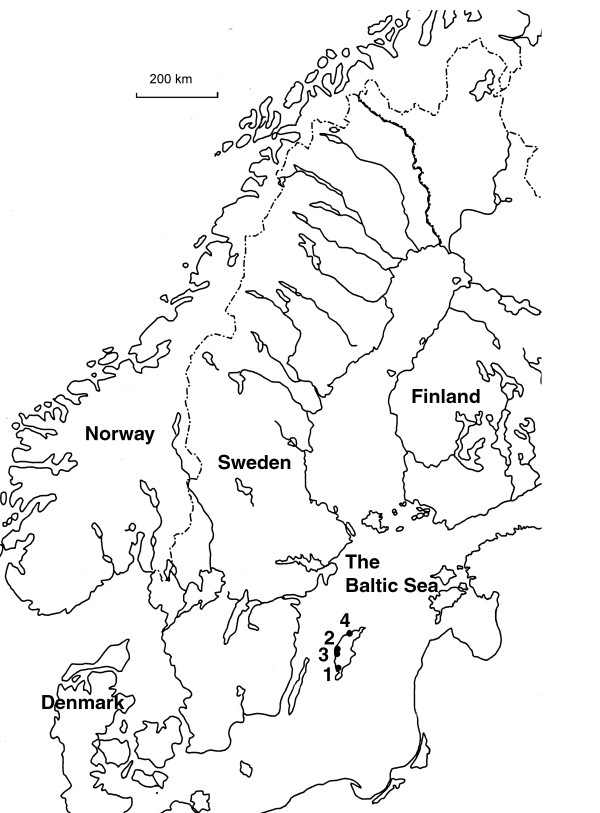
**Map of Scandinavia showing the archaeological sites**. 1. Ajvide, 2. Visby, 3. Fritorp, 4. Ire.

**Figure 2:**
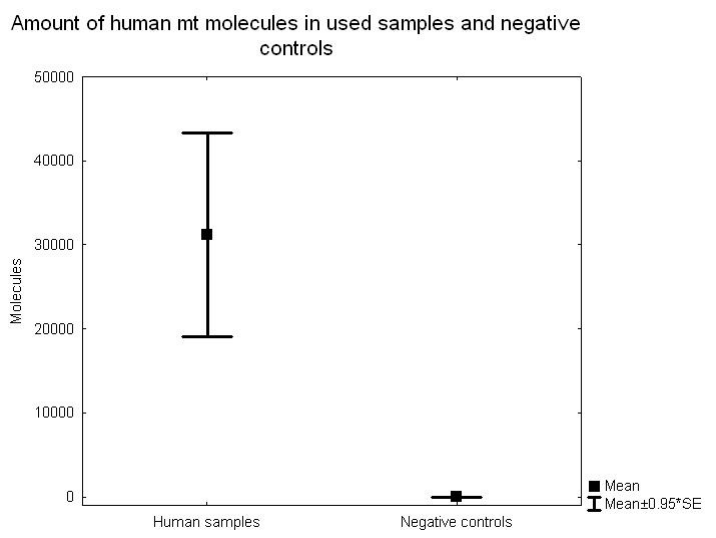
**Real-time PCR quantitative data for the 10 samples and 98 negative controls, where 31 were nonhuman mammals and 67 were water controls or PCR controls**.

The material consists of duplicate samples (teeth and/or ulna, femur or fibula) from 14 prehistoric individuals (Table 1). The samples originate from four archaeological sites on Gotland in the Baltic Sea dating to the Middle Neolithic, 4,800-4,200 BP (Figure 1). The 14 samples, originally from a larger set of 36 from Gotland and mainland Sweden, were chosen on the basis that they had all yielded high amounts of mitochondrial DNA compared to negative controls (Figure 2) [13]. The samples were collected from the following sites on Gotland: Ajvide (n = 9) in the parish of Eksta, Visby town (n = 1), Ire (n = 2) in the parish of Hangvar and Fridtorp (n = 2) in the parish of Västerhejde. All samples are at least a 1,000 years younger than the earliest evidence of agriculture in Scandinavia. Hence they represent an exclusively non-agricultural lifestyle considerably older than agriculture in the area. All of the samples are from burial contexts and were excavated, handled and stored following standard protocols for the excavation and storage of archaeological material. Thus, no special precautions were taken to minimize human DNA contamination. The nonhuman faunal samples, used as negative controls, were subject to the same excavation and storage protocols as the human samples, and thus would not be expected to yield a different contamination pattern compared to the human samples.

**Table 1:** Alleles at the polymorphic site -13910 in the lactase gene in 10 Middle Neolithic Scandinavian samples representing the Pitted Ware Culture (PWC).

Grave #	Site	C	C/T	T	Genotype	**Haplogroup **[13]
Grave 4	Ajvide	5			C	n.d
Grave 5	Ajvide	6			C	U5a
Grave 29A	Ajvide	7			C	U5a
Grave 36	Ajvide	5			C	U5
Grave 52A	Ajvide	4			C	V
Grave 70A	Ajvide	5			C	U4 or H1b
Grave 32	Visby	7			C	n.d
Grave 15	Fridtorp	5			C	U4 or H1b
Grave 3	Ire	4	2		C/T	U4 or H1b
Grave 8	Ire	4			C	U4 or H1b

DNA was extracted from approximately 100 mg of bone powder pre-treated with bleach [14]. The samples were extracted in a laboratory specially dedicated to aDNA work at the Archaeological Research Laboratory in Stockholm, as well as in a newly built ancient DNA facility at the National Board of Forensic Medicine in Linköping that is separated from the department's PCR areas. The samples were extracted at least twice, and both water blanks and extracts from contemporary prehistoric seals were included as negative controls. The DNA extractions were performed using the method of Yang *et al*. [15].

To screen for DNA content indicating authenticity, real-time PCR was performed on the selected samples as well as on 98 negative controls (where 31 were DNA extracts from ancient non human mammals representing the same context as the selected samples and 67 were water blanks). An 80 bp fragment in the mitochondria was targeted (L4567F 3'CACTGATTTTTTACCTGAGTAGGCCT5', H4595R 3'CGAGGGTTTATTTTTTTGGTTAGAACT5'), and amplifications were carried out according to a previously described protocol [14].

The C/T polymorphism at position -13910 upstream from the lactase gene was amplified by two different fragments, a shorter 53 bp fragment and a longer 168 bp fragment. The two sets of primers shared the biotinylated forward primer (5'→3' GCTGGCAATACAGATAAGATAATG), but had different reverse primers for the 53 bp fragment (5'→3'GAGGAGAGTTCCTTTGAGGC), and the 168 bp fragment (5'→3' ATGCCCTTTCGTACTACTCCC). The system was originally set up to detect differences in preservation and authenticity of the material analyzed, relying on the fragmentation level [14]. The PCR amplification was carried out using 5 μl of extract, 300 nM of each primer and the Illustra Hot Start Ready-To-Go mix (GE Healthcare Life Sciences). The PCR profile was 15 min at 95°C, followed by 43 cycles of 30 s at 94°C, 30 s at 54.6°C and 30 s at 72°C, with a final extension step of 15 min at 72°C. Pyrosequencing was used for allele identification. The sequencing primers were designed to anneal next to the SNP (5'→3'CCTTTGAGGCCAGGG). The pyrosequencing was performed according to supplier provided protocols, and as previously described in [16].

## Results

### Real-time PCR results

The 14 selected samples yielded over a 1000-fold higher concentration of human DNA (average 23,307 molecules) than the negative controls (average 18 molecules) [13]. When divided in non-human negative controls and water negative controls, the concentration of human DNA was still considerably lower in both cases compared to the 14 selected samples (non-humans 18 molecules, water blanks 18 molecules). When only considering the 10 samples yielding reproducible results, the concentration of human DNA also exceeded the negative controls by over a 1000 times (Figure 2).

### SNP results

The fourteen specimens all yielded replicable results and ten of these were successfully SNP-typed a minimum of four times (using both the 53 bp and the 168 bp fragment). Thus, even with thorough mitochondrial pre-selection, the success rate for the nuclear DNA sequences was down to 71%. As only one individual was heterozygote, it is not possible to calculate an allelic dropout rate, but from the four pyrosequencing results from the heterozygote individual, two replicate typing events expressed allelic dropout. A low number of positive results were detected in the negative controls, eight of the 59 Neolithic seals, seven of the 53 negative extraction controls and eight of the 47 PCR negative controls. In total, 14% of the negative controls were contaminated, and out of these, the frequency of the T allele was 52%. None of the contaminants could however be reproduced. The allele frequency in the negative controls was significantly different from the allele frequency in the ten archaeological samples used for further analysis (Fisher's Exact test, p < 0.001). The contamination frequency is in direct contrast to that of the short mitochondrial fragment, where 86% of the negative controls (98 negative controls, where 31 were nonhuman mammals and 67 water controls or PCR controls) expressed contamination, although to a more than 1000-fold lower quantity than the ancient human samples (Figure 2).

**Figure 3:**
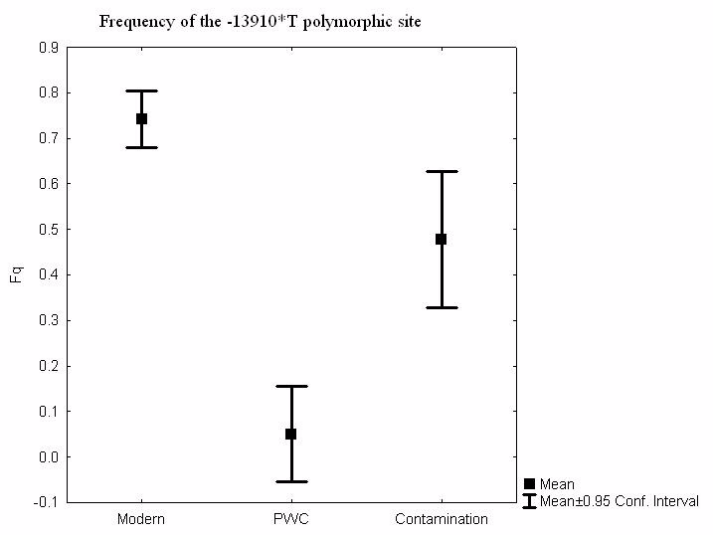
Frequency of the -13910*T polymorphic site in the lactase gene in three datasets: an extant Swedish population, a Swedish Neolithic hunter-gatherer population (PWC) and the negative controls.

Only one of the ten PWC individuals showed a presence of the T allele, a heterozygote, and the allele frequency for the T allele is 0.05 (1/20, with the exact 95% CI values 0.001265089 to 0.2487328, Figure 3). The T allele frequency in the PWC population differs significantly from the T allele frequency in the contemporary Swedish population (n = 97, Fisher's Exact test, p < 0.0001), where the frequency of the T allele is 0.74 (144/194, with the exact 95% CI values 0.6747198 to 0.8022533, Figure 3).

Genetic drift can cause differences in allele frequencies between two samples taken from the same population separated in time. Since the PWC and extant samples are separated by a substantial amount of time (around 4000 years), a simple Fisher's exact test would not account for genetic drift, but would only be relevant for the null-hypothesis of no difference between the two samples. To explore the possibility that genetic drift alone caused the difference in T allele frequency, we can model a prehistoric population (for example the PWC population) as being ancestral to a population existing today. To evaluate different demographic scenarios (e.g. constant population sizes or population expansion from the time of the prehistoric sample), we simulated an extant sample and a prehistoric sample using the program *COMPASS *[17]. *COMPASS *simulates different temporal samples under a coalescent model, and allows a range of demographic assumptions, for example, population expansion. For a particular assumption about the demographic model, we ran a large number of replicate simulations, and kept only samples that contained exactly 144 derived alleles (the T allele) in the extant sample. In other words, we conditioned on observing exactly 144 derived alleles in the extant sample as in the empirical modern data set. Out of 1,000 replicate simulations (which all had 144 derived alleles in the extant sample), we counted the number of times zero or one derived allele was observed in the prehistoric sample. This fraction can be used to approximate the probability of observing a prehistoric sample with zero or one derived allele, conditional on observing 144 derived alleles in the extant sample.

**Figure 4:**
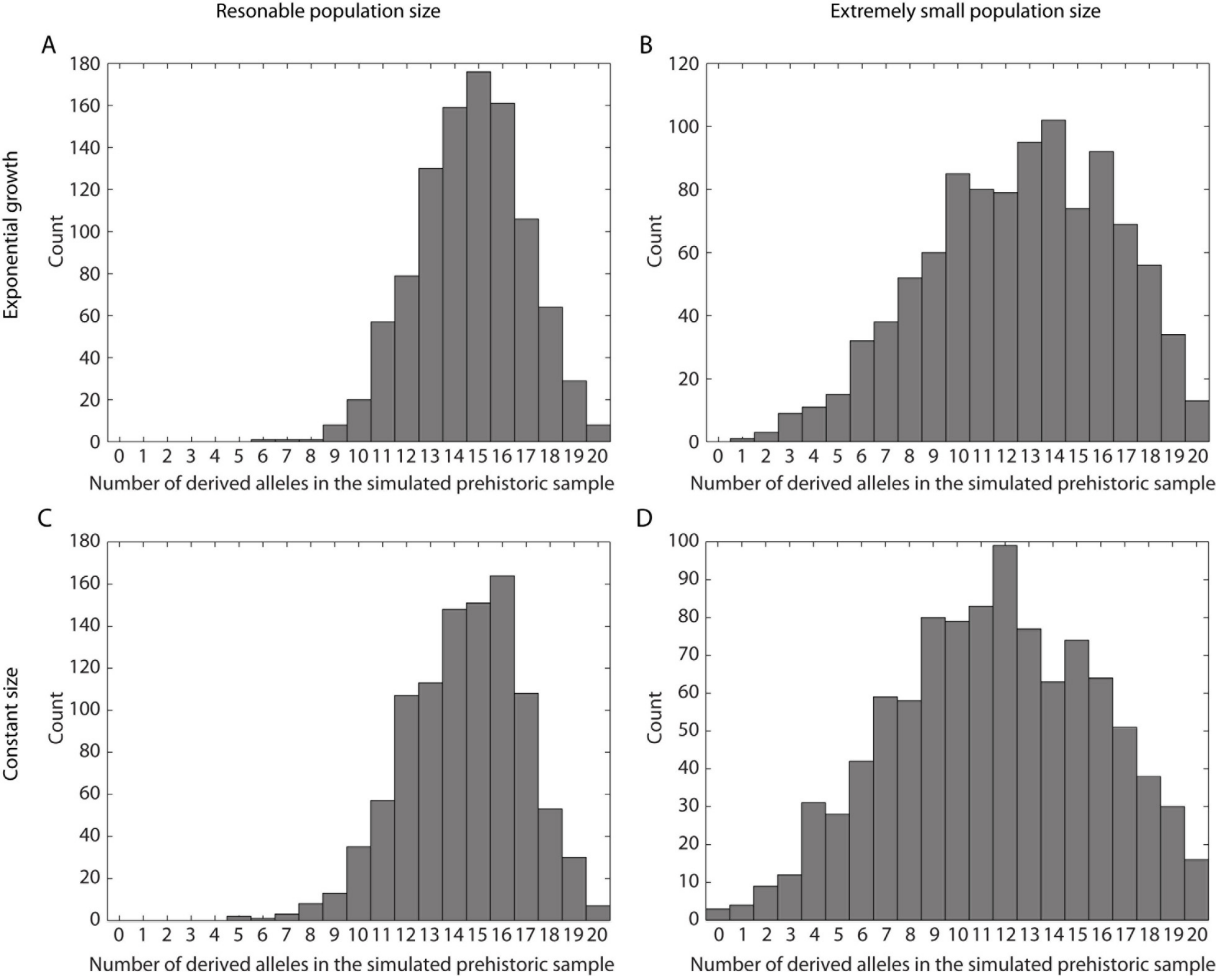
**Simulation results for four models where one prehistoric sample and one extant sample have been collected**. The distribution of a derived allele in the prehistoric sample, conditional on 144 derived alleles in the extant sample. A) The population starts to grow (exponentially) from 5,000 individuals 225 generations ago to a size of 50,000 individuals at present. B) The population starts to grow (exponentially) from 100 individuals 225 generations ago to a size of 10,000 individuals at present. C) Constant population size of 5,000 individuals. D) Constant population size of 500 individuals.

Our first scenario assumes that the effective population size in Sweden is 50,000 at present and that the population grew, exponentially, from *N*_*e *_= 5,000, starting 225 generations ago (before 225 generations ago, *N*_*e *_was constant and 5,000). We believe that this model may be a reasonable description of the demographic history of northern Europeans based on evidence from other European populations (see e.g. [18]), but it turns out that the results are robust to relatively small effective population sizes (see below). We simulate data for one SNP under an exponential growth model, with a contemporary sample size of 194 gene-copies and a size of 20 gene-copies in the prehistoric sample. By conditioning on observing 144 derived alleles in the contemporary sample, we retain 1,000 simulated data sets, and of these 1,000 sets, no simulation had zero or one derived allele in the prehistoric sample (see Figure 4A). If we decrease the population sizes in the simulation (and thereby increase the effect of genetic drift) we can determine how robust the result is to small population sizes. Assuming that the effective population size in Sweden is 10,000 at present and that the population grew, exponentially, from *N*_*e *_= 100, starting 225 generations ago (before 225 generations ago, *N*_*e *_was constant and 100), we found one simulated data set (out of 1,000) that had one derived allele in the ancient sample and no simulated data set that had zero derived alleles in the prehistoric sample. Figure 4B shows the distribution of the number of derived alleles in the prehistoric sample (conditional on 144 derived alleles in the extant sample).

To conclude, for a neutral site (a SNP) in an unstructured population, we note that, 1) assuming a constant population size (Figure 4C), or an exponentially growing population (Figure 4A), and for reasonable assumptions about the population sizes, the probability is very small of observing the configuration of one or no derived allele (out of 20) in the prehistoric sample and 144 derived alleles (out of 194) in the extant sample. 2). Even for extreme population histories, such as a constant population of 100 individuals until 225 generations ago, and where the population grew exponentially to 10,000 individuals (Figure 4B), or a constant size of 500 individuals (Figure 4D), the probability of observing one or zero derived alleles in the prehistoric sample is still small. Thus, it is unlikely that genetic drift caused the observed frequency in the PWC given the frequency in the extant Swedish population. This leaves either selection or differences in population structure between the prehistoric sample and the extant sample (i.e., the PWC is not an ancestral population to the extant Swedish population) as possible explanations.

## Discussion

Authentication of DNA sequences in prehistoric human remains has proved complicated, mainly because modern human contamination is always present to some degree [19-21]. Here we rely on a combination of several arguments for our acceptance of the results as authentic [21-23]. The most important argument is that the success rate, like the allele frequencies, differs among the prehistoric human remains, the present-day human samples and the negative controls. Further, our material had already been pre-screened for contamination, including a major contamination investigation in which human and nonhuman DNA was typed in large parallel series of human and animal assemblages from the same sites (Figure 3) [24].

The frequency of the derived T allele, strongly associated with the ability to consume unprocessed milk at adulthood, was significantly different between the prehistoric and the extant samples. For reasonable assumptions about population sizes our simulations show that it is highly unlikely that the frequency could have shifted over time due to drift in a population with constant size, or in an expanding population. Thus, only strong positive selection or differences in population structure between the PWC population and the population ancestral to the extant Swedish population would explain the discrepancy between the extant and the prehistoric data. We note that with the data on a single SNP presented here, it is not possible to discriminate between these two scenarios. Also, it should be noted that the prehistoric samples used in this study came from a non-agricultural population, existing in parallel with an agricultural population (TRB) that is potentially ancestral to the extant Northern European population [13]. In Burger et al. [11] one Mesolithic, nine Neolithic and one Medieval samples were analyzed. In this study 9 of the 10 individuals were homozygous C at position -13.910 only the medieval individual was heterozygous for the -13.910-C/T polymorphism. These data show some similarity with our data as the Mesolithic individual does not show any lactase persistence.

Here we describe a possible scenario where cultural practices could have had a tremendous impact on the genetic composition of human populations. Before dairy farming was practiced in Scandinavia, the allele frequency at -13910 was not greatly affected. The neolithisation processes in Scandinavia provided a selection pressure on this specific locus on a scale that may actually have led to a population replacement in the area (as indicated by mitochondrial DNA [13]), or at least a major shift in the allele frequency at this particular locus. This would require that cultural changes were dependent upon demographic changes in this case, and that cattle farming presented a clear advantage to hunting/gathering. In such case the 'secondary product revolution' (i.e. the introduction of milk) in northern Europe would have a large effect on the derived allele at -13910. Thus, the development that led to the present-day North European lifestyle, which is heavily based on dairy and other farm products, may have been a process where cultural practices and genes interacted.

## Conclusion

It is unlikely that the PWC and their ancestors used dairy products before the middle Neolithic, nor is there any archaeological evidence for such practices [25]. The difference in allele frequency from the extant Scandinavian population may therefore be explained by the fact that PWC is not ancestral to the extant Scandinavian population. An alternative, but less likely, explanation posits an ancestral relationship, but with selection dramatically increasing the frequency of the derived allele.

## Authors' contributions

AL, HM, and AG conceived, designed the project, performed the experiments and analyzed the data. MJ designed the bioinformatic part of the project and analyzed the data. JS and PM supplied the samples and performed osteological analysis. AL, HM, AG, MJ, GH and KL helped to draft the manuscript. All authors read and approved the final manuscript.

## References

[B1] McCracken RD Lactase Deficiency: An Example of Dietary Evolution Current Anthropology 1971 12 479 10.1086/201234

[B2] Holden C Mace R Phylogenetic analysis of the evolution of lactose digestion in adults Human Biology 1997 69 605 628 9299882

[B3] Bellwood P First Farmers The Origins of Agricultural Societies 2005 Malden: Blackwell Publishing

[B4] Beja-Pereira A Caramelli D Lalueza-Fox C Vernesi C Ferrand N Casoli A Goyache F Royo LJ Conti S Lari M Martini A Ouragh L Magid A Atash A Zsolnai A Boscato P Triantaphylidis C Ploumi K Sineo L Mallegni F Taberlet P Erhardt G Sampietro L Bertranpetit J Barbujani G Luikart G Bertorelle G The origin of European cattle: Evidence from modern and ancient DNA Proc Natl Acad Sci USA 2006 103 8113 8 10.1073/pnas.0509210103 16690747 PMC1472438

[B5] Bersaglieri T Sabeti PC Patterson N Vanderploeg T Schaffner SF Genetic signatures of strong recent positive selection at the lactase gene Am J Hum Genet 2004 74 1111 1120 10.1086/421051 15114531 PMC1182075

[B6] Tishkoff SA Reed FA Ranciaro A Convergent adaptation of human lactase persistence in Africa and Europe Nat Genet 2007 39 31 40 10.1038/ng1946 17159977 PMC2672153

[B7] Kuokkanen M Butzow R Rasinperä H Medrek K Nilbert M Malander S Lubinski J Järvelä I Lactase persistence and ovarian carcinoma risk in Finland, Poland and Sweden International Journal of Cancer 2005 117 90 94 10.1002/ijc.21130 15880573

[B8] Enattah NS Sahi T Savilahti E Terwilliger JD Peltonen L Jarvela I Identification of a variant associated with adult-type hypolactasia Nat Genet 2002 30 233 237 10.1038/ng826 11788828

[B9] Coelho M Luiselli D Microsatellite variation and evolution of human lactase persistence Human Genetics 2005 117 329 339 10.1007/s00439-005-1322-z 15928901

[B10] Itan Y Powell A Beaumont MA Burger J Thomas MG The Origins of Lactase Persistence in Europe PLoS Comput Biol 2009 5 e1000491 10.1371/journal.pcbi.1000491 19714206 PMC2722739

[B11] Burger J Kirchner M Bramanti B Haak W Thomas MG Absence of the lactase-persistence-associated allele in early Neolithic Europeans Proc Natl Acad Sci USA 2007 104 3736 3741 10.1073/pnas.0607187104 17360422 PMC1820653

[B12] Malmer M The Neolithic of south Sweden: TRB, GRK, and STR 2002 Royal Swedish Stockholm: Academy of Letters, History and Antiquities, Almqvist & Wiksell International

[B13] Malmström H Gilbert MT Thomas MG Brandström M Storå J Molnar P Andersen PK Bendixen C Holmlund G Götherström A Willerslev E Ancient DNA reveals lack of continuity between neolithic hunter-gatherers and contemporary Scandinavians Curr Biol 2009 19 1758 62 10.1016/j.cub.2009.09.017 19781941

[B14] Malmström H Svensson EM Gilbert MTP Willerslev E Götherström A Holmlund G More on Contamination: The Use of Asymmetric Molecular Behavior to Identify Authentic Ancient Human DNA Mol Biol Evol 2007 24 998 1004 10.1093/molbev/msm015 17255122

[B15] Yang DY Eng B Waye JS Dudar JC Saunders SR Improved DNA extraction from ancient bones using silica-based spin columns American Journal of Physical Anthropology 1998 105 539 543 10.1002/(SICI)1096-8644(199804)105:4<539::AID-AJPA10>3.0.CO;2-1 9584894

[B16] Anderung C Bouwman A Persson P Carretero JM Ortega AI Elburg R Smith C Arsuaga JL Ellegren H Götherström A Prehistoric contacts over the Straits of Gibraltar indicated by genetic analysis of Iberian Bronze Age cattle PNAS 2005 102 8431 8435 10.1073/pnas.0503396102 15941827 PMC1150856

[B17] Jakobsson M COMPASS: A program for generating serial samples under an infinite sites model Bioinformatics 2009 25 2845 10.1093/bioinformatics/btp534 19762347

[B18] Tenesa A Navarro P Hayes BJ Duffy DL Clarke GM Goddard ME Visscher PM Recent human effective population size estimated from linkage disequilibrium Genome Research 2007 17 520 526 10.1101/gr.6023607 17351134 PMC1832099

[B19] Haak W Forster P Bramanti B Matsumura S Brandt G Tanzer M Villems R Renfrew C Gronenborn D Alt KW Burger J Ancient DNA from the First European Farmers in 7500-Year-Old Neolithic Sites Science 2005 310 1016 1018 16284177 10.1126/science.1118725

[B20] Sampietro ML Gilbert MT Lao O Caramelli D Lari M Bertranpetit J Lalueza-Fox C Tracking down human contamination in ancient human teeth Mol Biol Evol 2006 23 1801 7 10.1093/molbev/msl047 16809622

[B21] Malmström H Stora J Dalen L Holmlund G Gotherstrom A Extensive Human DNA Contamination in Extracts from Ancient Dog Bones and Teeth Mol Biol Evo 2005 22 2040 2047 10.1093/molbev/msi195 15958782

[B22] Richards MB Sykes BC Hedges REM Authenticating DNA Extracted From Ancient Skeletal Remains Journal of Archaeological Science 1995 22 291 299 10.1006/jasc.1995.0031

[B23] Kolman CJ Tuross N Ancient DNA analysis of human populations American Journal of Physical Anthropology 2000 111 5 23 10.1002/(SICI)1096-8644(200001)111:1<5::AID-AJPA2>3.0.CO;2-3 10618586

[B24] Linderholm A Malmström H Lidén K Holmlund G Götherström A Cryptic Contamination and Phylogenetic Nonsense PLoS ONE 2008 3 e2316 10.1371/journal.pone.0002316 18509458 PMC2384008

[B25] Eriksson G Linderholm A Fornander E Kanstrup M Schoultz P Olofsson H Lidén K Same island, different diet: Cultural evolution of food practice on Öland, Sweden, from the Mesolithic to the Roman Period Journal of Anthropological Archaeology 2008 27 520 543 10.1016/j.jaa.2008.08.004

